# Detection and determination of stability of the antibiotic residues in cow’s milk

**DOI:** 10.1371/journal.pone.0223475

**Published:** 2019-10-10

**Authors:** Mahantesh Kurjogi, Yasser Hussein Issa Mohammad, Saad Alghamdi, Mostafa Abdelrahman, Praveen Satapute, Sudisha Jogaiah

**Affiliations:** 1 Laboratory of Plant Healthcare and Diagnostics, Department of Studies in Biotechnology and Microbiology, Karnatak University, Dharwad, Karnataka, India; 2 Department of Biochemistry, Applied Science college, Hajjah University, Hajjah, Yemen; 3 Laboratory Medicine Department, Faculty of Applied Medical Sciences, Umm Al-Qura University, Makkah, Saudi Arabia; 4 Arid Land Research Center, Tottori University, Tottori, Japan; Purdue University, UNITED STATES

## Abstract

In the present study, antibiotic residues were detected in milk samples collected from the dairy herds located in Karnataka, India, by microbiological assay. Subsequently, the detected antibiotics were identified as azithromycin and tetracycline, by high-performance liquid chromatography, further both the antibiotics detected in the cow milk samples were found to be at high concentration (9708.7 and 5460 μg kg^-1^, respectively). We then investigated the effects of temperature and pH on the stabilities of azithromycin and tetracycline to determine the degradation rate constant *k* using first-order kinetic equation. Results indicated that significant reduction in stability and antibacterial activity of azithromycin solution when subjected to 70 and 100°C for 24 h. While stability of tetracycline was significantly reduced when subjected to 70 and 100°C for 24 h. However no significant reduction in antibacterial activity of tetracycline was observed at respective temperatures when compared with that of control. In addition, the stabilities of azithromycin and tetracycline were found to be decreased in acidic pH 4–5. The results of the present study revealed the high risk of contamination of milk sample with veterinary antibiotics and also demonstrated the effect of temperature and pH on stability of antibiotics. Therefore the study suggest that the qualitative and quantitative screening of milk for the presence of antibiotics need to be strictly performed to ensure safe drinking milk for consumers.

## Introduction

Cow milk, being the richest natural source for nutritional elements, is the most commonly consumed milk in the world [1,2]. However, the cow milk currently available in the market is laced with chemical residues like pesticides and antibiotics. The presence of antibiotic residues in milk not only affects its quality but also constitutes a significant health hazard to the consumers [3]. In most conventional dairies, cows are regularly treated with antibiotics as a preventive measure, and intramammary injection has been proven to be an effective mode of treatments for udder infections [4]. However, the excessive use of antibiotics against intramammary infections in dairy herds affects the quality of milk produced, and subsequently consumers health, due to the presence of the antibiotic residues in milk [5–7]. Such milk may provoke allergies, intestinal alterations and emergence of multidrug-resistant bacteria among consumers [8]. Antibiotic residues may also inhibit the normal microflora of milk, and have an adverse effect on the manufacturing processes of some dairy products, such as yogurt and cheese [1,9,10]. In addition, lack of information on the withdrawal period and the stability of antibiotic residues leads to failure in controlling excessive use of veterinary drugs [11,12]. Some veterinary drugs like tetracycline are reabsorbed through entero-hepatic circulation and persisted in the body for a long time after administration, resulting in photosensitivity reaction with developed pigmentation of the nails [13]. Consumption of tetracycline-contaminated milk for short or long duration may lead to the developments of permanent discoloration of the teeth in children [14]. Similarly, drinking milk contaminated with high levels of azithromycin can severely affect the human immune and cardiovascular system [15].

India is the world’s largest milk producer with 18 percent global production (Retrieved December 14^th^ 2018, from http://www.fao.org/dairy-production-products/production/en/). The farms in India have been routinely using antibiotics, particularly azithromycin and tetracycline, in cattle husbandry practices [10,16,17]. The occurrence of antibiotic residues in market milk has been frequently reported in India, raising a great concern among the consumers [10,16,18]. The usage of antibiotics varies from region to region and among individual cows within farms, depending on the causative agent and the severity of illness. Hence, it is essential to detect the prevalence of antibiotic residues in milk of each particular region, and perhaps for individual cows within farms, for formulating the proper regional guidelines to ensure safe milk for the consumers. Therefore, screening for antibiotic residues in milk before it reaches the consumers is critical in dairies as it will help to reduce the threat of residual contamination of the food chain. However the proper choice of antibiotic screening test plays an important role in effectiveness and accuracy of detection of antibiotic residues [19]. Various techniques like chemical, microbiological and immunological assays are available to detect antibiotic residues in milk, but most of these methods lack specificity, as they provide only semi-quantitative measurements and produce false positive results [20]. To overcome these problems, chromatographic techniques, such as high-performance liquid chromatography (HPLC), was developed and known to be the most reliable, sensitive and robust method [21–23]. Available litreture showed that, HPLC was able to determine sulfonamides from food samples [24] and also HPLC method was successfully used to detect the antibiotic residues at lower than their respective maximum residue limits values [25]. Thus, in the present study the microbiological assay was first carried out to detect the presence of antibiotic residues in the cow milk samples collected from different farms in Dharwad district of Karnataka, India. Subsequently, the HPLC was used for identification and quantification of the antibiotic residues present in milk samples. Finally, the effects of temperature and pH on stability of antibiotics were evaluated with respect to their antimicrobial activity.

## Materials and methods

### Chemicals and reagents

Acetonitrile and methanol of HPLC-grade, as well as azithromycin and tetracycline were procured from S D Fine-Chem, Mumbai, India. Millipore water was used in all analyses. Media for antibacterial activity, sterile paper disc and zone inhibition measuring scale were procured from HiMedia.

### Collection of milk samples

A total of 13 raw cow milk samples (100 mL per sample) were collected from the dairy herds located in the Dharwad district of Karnataka, India, using sterilized container. Samples were stored at 4°C until analyses. No animal research was carried out in the study and also we declare that no animals were sacrificed or anesthesized in the present study.

### Antimicrobial assay of milk samples

*Bacillus subtilis* culture was grown in Brain-Heart infusion liquid medium at 37°C. After 6 h of growth, 0.1 mL of broth (10^6^ cells mL^-1^) was spread on the surface of Mueller-Hinton agar plates. Sterile paper discs of 10 mm size were entirely dipped in 100 mL of milk sample using forceps until the discs were completely impregnated with the milk sample. Milk-wetted discs were air-dried and placed equidistantly around the margin of the inoculated plates. Discs dipped into sterile distilled water (SDW) and pasteurized packet milk were served as control 1 and 2 respectively. Plates were incubated at 37 ± 2°C for 24 h, and zone showing inhibition of the bacterial growth was measured in mm using zone inhibition scale. Each test was performed in triplicate.

### Preparation of milk samples for HPLC

Milk samples collected from dairy herds were processed as previously described by Khaskheli et al. with some modifications. 100 mL of raw milk sample were treated with 8 mL of 10% aqueous solution of acetic acid, and the mixture was then centrifuged at 3500 rpm for 10 min at 4°C [26]. The clear supernatant was taken by a disposable syringe without disturbing the fat layer, and was filtered through a 0.45-μm nylon filter. Subsequently, the filtered extract (1 mL) was transferred to a 2 mL sterilized vial and used for HPLC to identify azithromycin and tetracycline residues.

### Quantification of azithromycin and tetracycline residues by HPLC

Ten μL of extract were injected into an HPLC Jasco device equipped with variable wavelength UV detector coupled with a C-18 column (octadecyl silyl, 100Å, 250 × 4.6 mm, 5 μm) at 214 nm and 355 nm for quantification of azithromycin and tetracycline, respectively. Elution was carried out at a flow rate of 1.2 mL min^-1^ using the isocratic mobile phase consisted of water:acetonitrile:methanol (55:25:20, v:v:v) for azithromycin, and methanol:acetonitrile:aqueous oxalic acid (1:2:4.5, v:v:v) for tetracycline. Azithromycin and tetracycline standard solutions were prepared by accurately weighing 1 mg of azithromycin and tetracycline and transferred to 50 ml HPLC grade water in volumetric flask. An aliquot of 5.0 ml was further diluted with HPLC grade water in 100 ml volumetric flask, to obtain a final concentration of 50 μg mL^-1^and store at 4°C. Serial dilutions (5, 10, 15 and 20 μg mL^-1^) were injected and eluted as mentioned above. The quantification of the antibiotic residues was achieved by comparison of the peak area of the sample with that of the standard having the same chromatogram.

### Degradation kinetics of azithromycin and tetracycline

One mg mL^-1^ azithromycin or tetracycline was prepared in 0.2 M phosphate buffer (Stock solutions of monobasic and dibasic were prepared by dissolving 27.6 and 28.4 g of monobasic sodium phosphate, monohydrate and dibasic sodium phosphate respectively in 1000 ml distilled water and stored at 4°C. Phosphate buffer of 0.2 M was obtained by mixing 39 ml of dihydrogen sodium phosphate with 61 ml of disodium hydrogen phosphate), and maintained in a thermostat at definite temperatures of 4, 37, 70 and 100°C for different time intervals (0, 1, 3, 6, 12 and 24 h). A series of trisodium citrate buffer solutions of 0.06 M with a different pH range of 4.0–9.0 containing 1 mg mL^-1^ of azithromycin or tetracycline were prepared and kept at 24°C for different time intervals (0, 1, 3, 6, 12 and 24 h). The deviation of azithromycin and tetracycline concentrations due to the effects of different temperatures and pHs was measured by a Hitachi 2500 spectrophotometer at 214 nm and 355 nm, respectively. The resultant data were inserted into the first-order kinetic equation below to determine the antibiotic degradation rate constant *k*:

ln([Ct]/[Ci])=−kt(1)

where *Ci* is the initial concentration of the antibiotic at time 0, *Ct* is the concentration of the antibiotic at time *t* and kt is antibiotic degradation rate constant at time t.

### Evaluation of antibacterial activities of azithromycin and tetracycline at different temperature

Sterile paper discs were soaked into samples, each had 20 μL phosphate buffer containing 1 mg mL^-1^ of azithromycin or tetracycline, which were maintained at different temperatures (4, 37, 70 and 100°C) for 24 h. Antibiotic solution-wetted paper discs were air-dried and placed on the surface of inoculated Mueller-Hinton agar medium containing *B*. *subtilis* (10^6^ CFU mL^-1^) prepared as described above. Sterile discs saturated with 20 μL of freshly prepared antibiotic solution or 20 μL of buffer alone were simultaneously used as positive and negative controls, respectively. The plates were incubated at 37°C for 24 h, and inhibition zone was measured in the cultured plate in mm.

## Results and discussion

### Antibiotic residues in the collected cow milk samples

**Fig 1:**
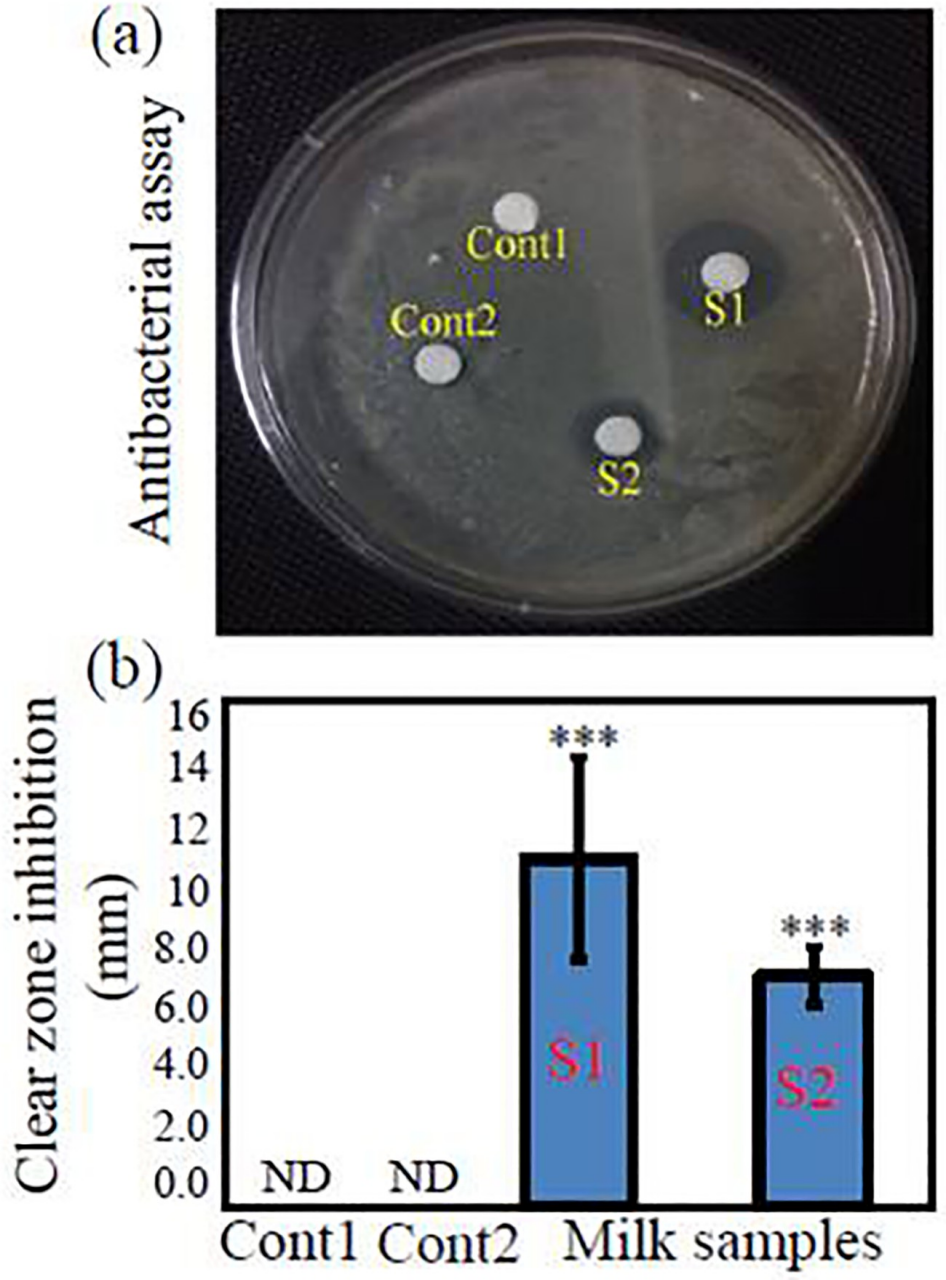
Antibacterial assay of the raw milk samples S1 and S2 against *Bacillus subtilis* using paper disc agar diffusion method. (a) Antibacterial plate assay and (b) clear zone inhibition in mm. Values are means ± standard errors of three independent replicates (*n* = 3). Significantly difference at *P* < 0.05 was determined using analysis of variance. ND, not detected; Cont 1, disc dipped in sterile distilled water; Cont 2, disc dipped in pasteurized packet milk.

Testing milk and its products for antibiotic residues is now a part of the routine quality control. All 13 raw milk samples were subjected to an antibacterial assay, out of which two milk samples (S1 and S2) showed a significant (*P* < 0.001) inhibition of the bacterial growth as compared with control (Fig 1). The positive result of the antibacterial assay indicated the presence of antibiotic residues in the tested raw milk samples (Fig 1). Similarly, Khaskheli et al. [26] detected antibiotic residues in the milk available at the market of Pakistan where *B*. *subtilis* was used as a test organism using field disc assay [26]. Movassagh and Karami (2010) also determined the presence of antibiotic residues in bovine milk of Tabriz, Iran [27]. Additionally, a study conducted in Parsbad, Iran, also showed that 14% of the raw cow milk samples were positive in antibiotic residues [28]. Similarly, Padol et al. reviewed that inappropriate uses of antibiotics in the treatment of the animal diseases may lead to the appearance of antibiotic residues in milk, which postures the risk of human health hazards and also interferes with the processing of milk products [18]. The results of the present study revealed that the antibiotic residue contents in the tested milk samples were high enough to inhibit the growth of the tested bacterium (Fig 1), which could also kill the normal microflora of milk that serves as a starter culture in the preparation of fermented milk products [18]. The antibiotics in the milk might also alter the probiotic concentrations through antibiosis, leading to harmful health effects [29].

### Contamination levels of azithromycin and tetracycline residues in the examined cow milk samples

**Fig 2:**
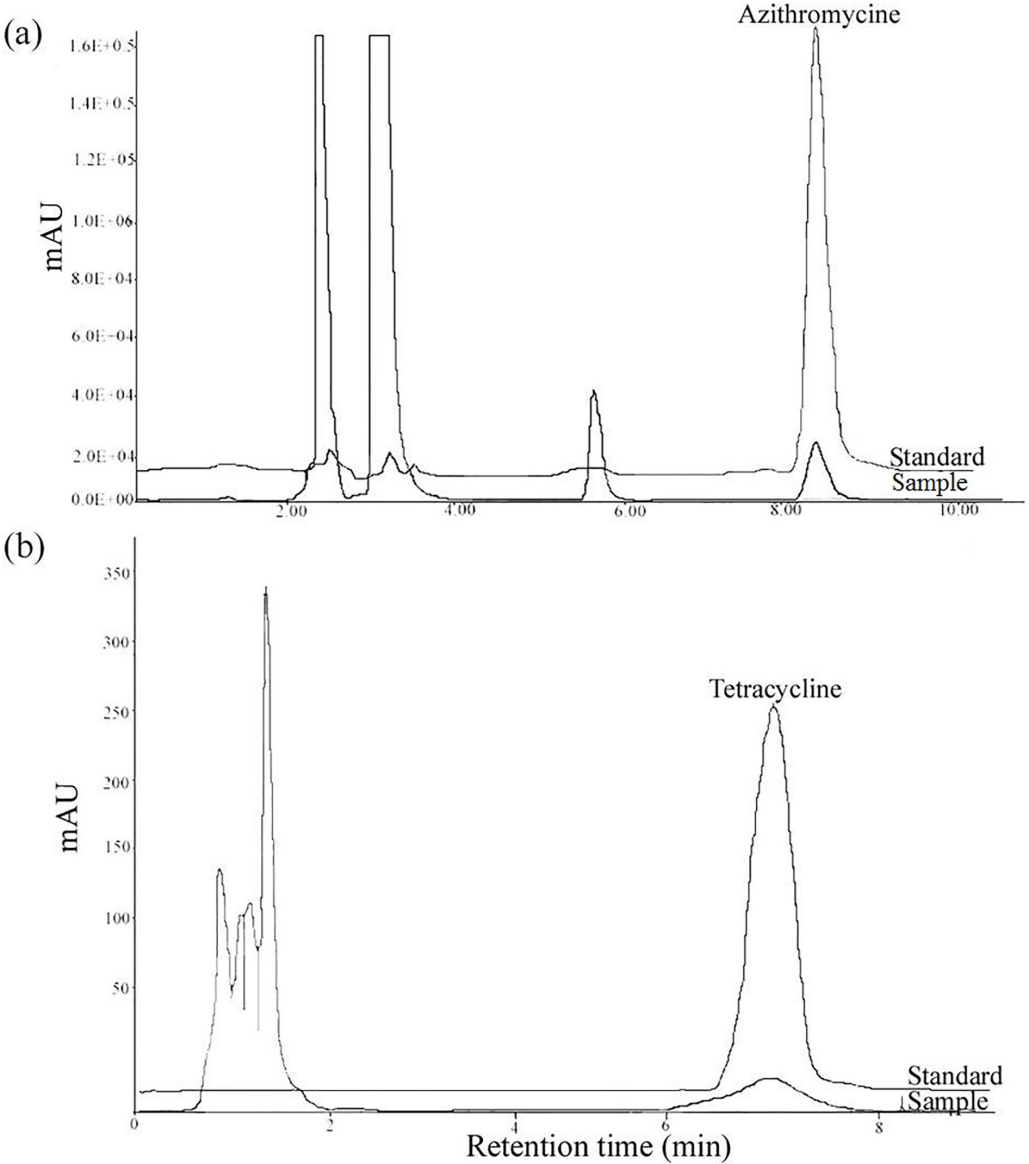
Identification and quantification of antibiotic residues in S1 and S2 raw milk samples using high-performance liquid chromatography (HPLC). (a and b) HPLC chromatogram of S1 and S2 milk samples correlated with authentic standard.

Based on the previously published literature and information obtained from the farmers of the examined areas, three antibiotics viz azithromycin, tetracycline and penicillin were suspected and further screening revealed that penicillin residue was not detected in our milk samples. Whereas two milk samples were found to be contaminated with azithromycin and tetracycline residues. We knew that azithromycin and tetracycline antibiotics are commonly used in animal husbandry practices [15,10,18]. however, the levels of contaminations and stabilities of these two antibiotics remained elusive. To verify this hypothesis, we used azithromycin and tetracycline antibiotics as standard controls in an HPLC to detect and quantify these antibiotic residues in the two contaminated milk samples (S1 Fig). Indeed, the peak signals detected from the milk samples at 214 nm and 355 nm matched well to those of the authentic controls, verifying the presence of azithromycin and tetracycline respectively as antibiotic residues in these two milk samples. Similarly after complete run of HPLC azithromycin standard and S1 samples were eluted at 8.5 mins. Alike, the sample analyzed for tetracycline was eluted at 7.1 mins which was corresponding to its standard. (Fig 2a and 2b). The antibiotic residues of azithromycin and tetracycline in the S1 and S2 milk samples were found to be 9708.7 and 5460.0 μg Kg^-1^, respectively. Quantification analysis indicated that the detected antibiotic levels were found to be remarkably higher than the maximum residue limit for antibiotic residues in milk set by Food Drug Administration (FDA) of the United States and European Union [30] [http://eur-lex.europa.eu/LexUriServ/LexUriServ.do?uri=OJ:L:2009:152:0011:0022:en:PDF; http://eur-lex.europa.eu/legalcontent/EN/ALL/?uri=CELEX%3A32002D0657].

Available reports suggest that large amount of azithromycin and other macrolide residues present in milk not only affect the quality of milk but also cause serious changes in the function of cardiac contraction [31], and sometimes may result in permanent hearing loss or vertigo in humans [32]. The rate of metabolism of tetracycline in cows has been estimated to 25–75%, and a significant percentage of the administrated tetracycline is excreted in bovine milk [33]. The antibiotics and their metabolites may end up in milk and may cause harmful effects to consumers, if the withdrawal period of antibiotics has not been passed [34]. Penicillin G residue was reported in raw cow milk in Pakistan and Venezuela [35,26]. Erskine et al., (1995) also observed longer excretion of ceftiofur in the milk of cows with induced *Escherichia coli* mastitis when compared with healthy cows [36]. Polymyxin B was not efficiently absorbed after intramammary treatment of healthy cows, or from cows with chronic mastitis, and 90% of the applied amount of polymyxin B were later found in the milk [37]. The detection of antibiotic residues in the milk revealed that the antibiotics used for the treatment of intramammary infection were still persisted in the cows at the time of milking, indicating the lack of knowledge of antibiotic withdrawal period. Therefore, the results from our study suggest that the cows treated with an antibiotic should not be milked before the complete withdrawal of the antibiotic used in treatment.

### Thermal kinetics of azithromycin and tetracycline

Several studies investigated the effects of temperature on stability of antibiotics and presented their findings in terms of degradation of antibiotic residues or reduction of antimicrobial activity in the food product in response to thermal treatment. However the results of degradation of antibiotics reported in available litreture vary depending upon the selection of temperature for treatment, the solvent and the pH. The thermal kinetics of azithromycin and tetracycline were investigated, and the antibiotic degradation rate constant *k* was summarized in Tables 1 and 2. The degradation rate constant *k* of azithromycin at 4°C was 1000 ± 8.10^−4^ after 1 h, which later drastically decreased to 62 ± 2.10^−4^ after 24 h (Table 1). On the other hand, at 37°C the azithromycin constant *k* sharply increased from 50 ± 1.10^−4^ to 966 ± 9.10^−4^ after 3 h of incubation, then gradually decreased (Table 1). In addition, a significant increase in the degradation rate constant *k* from 50 ± 2.0^−4^ to 4033 ± 22.10^−4^ was recorded at 70°C after 3 h of incubation, which drastically decreased to 537 ± 5·10^−4^ after 24 h incubation (Table 1). Likewise, changes in constant *k* at 100°C were recorded revealing a significant increase from 200 ± 3.10^−4^ to 3900 ± 18.10^−4^ after 3 h of incubation, followed by a gradual decrease (Table 1).

**Table 1:** Azithromycine degradation constant rate *k* values at different temperatures.

“*k* (10^−4^)”				
Time (hour)	4°C	37°C	70°C	100°C
1	1000 ± 8^e^	50 ± 1^a^	50 ± 2^a^	200 ± 3^a^
3	333 ± 3^d^	966 ± 9^d^	4033 ± 22^e^	3900 ± 18^e^
6	250 ± 2^c^	933 ± 8^d^	2000 ± 15^d^	2033 ±16^d^
12	116 ± 2^b^	516 ± 7^c^	1025 ± 8^c^	1000 ± 6^c^
24	62 ± 2^a^	270 ± 4^b^	537 ± 5^b^	533 ± 4^b^

Different letters indicate statistically significant differences at *P* < 0.05 according to a Tukey’s honest significant difference *post hoc test*. Values are mean ± standard errors of triplicate samples.

**Table 2:** Tetracycline degradation constant rate *k* values at different temperatures.

“*k* (10^−4^)”				
Time (hour)	4°C	37°C	70°C	100°C
1	20 ± 4^a^	50 ± 6^a^	30 ± 6^a^	60 ± 15^a^
3	256 ± 32^e^	400 ± 46^e^	630 ± 81^e^	860 ± 61^e^
6	163 ± 17^cd^	290 ± 23^cd^	360 ± 17^cd^	510 ± 46^d^
12	116 ± 26^c^	225 ± 12^c^	300 ± 26^c^	340 ± 32^c^
24	50 ± 8^b^	135 ± 20^b^	160 ± 18^b^	160 ± 02^b^

Different letters indicate statistically significant differences at *P* < 0.05 according to a Tukey’s honest significant difference *post hoc test*. Values are mean ± standard errors of triplicate samples.

**Fig 3:**
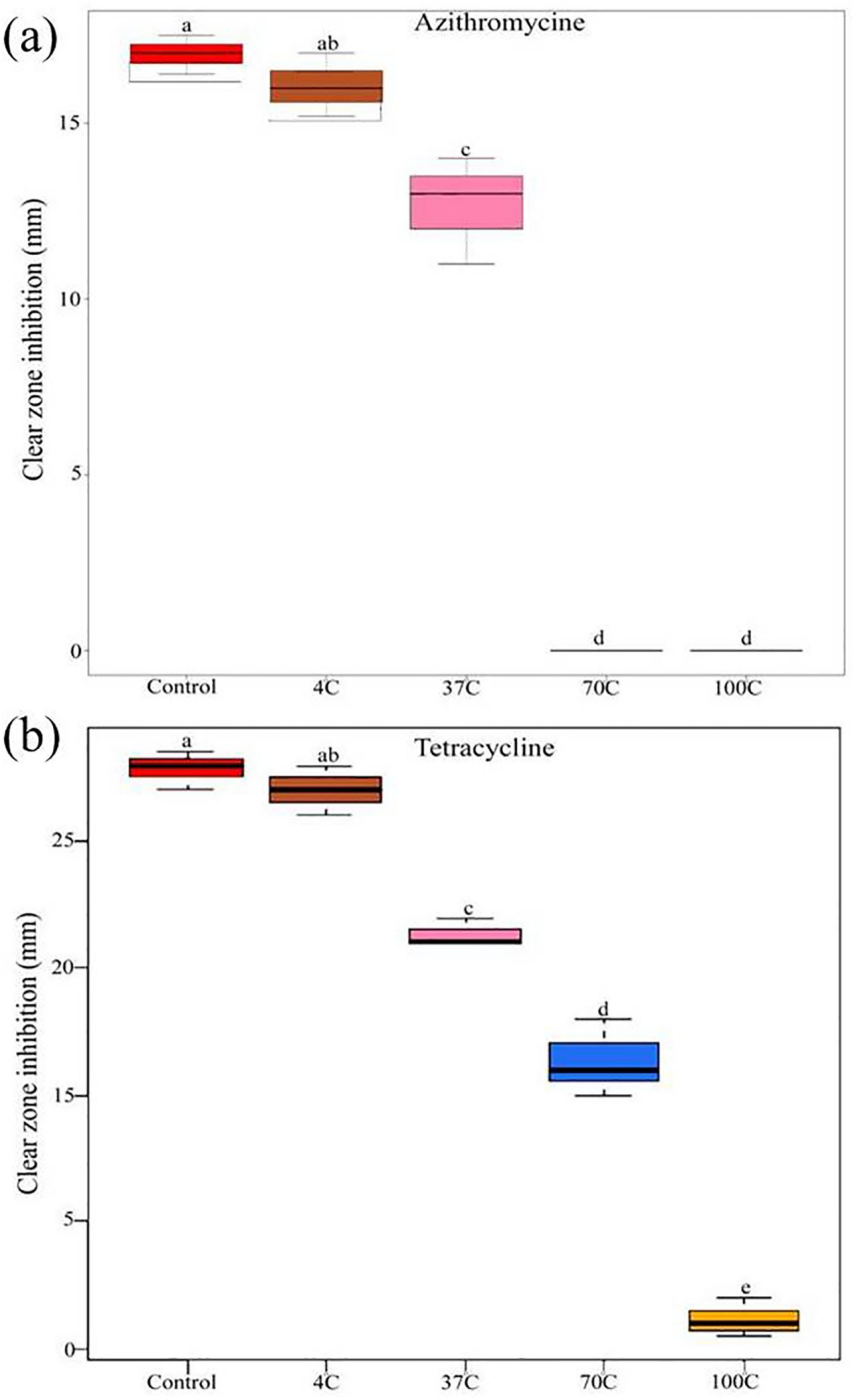
Box plot showing changes in (a) azithromycine and (b) tetracycline antibacterial activities at different temperature treatments against *Bacillus subtilis*. Values represent the maximum, third quartile, median, first quartile and minimum of three independent replicates (*n* = 3). Different letters indicate statistically significant differences at *P* < 0.05 according to a Tukey’s honest significant difference *post hoc test*.

The degradation rate constant *k* of tetracycline at 4°C was 20 ± 4.10^−4^ after 1 h, which remarkably increased to 256 ± 32.10^−4^ after 3 h of incubation, and then gradually decreased to 50 ± 8.10^−4^ at 24 h of incubation (Table 2). At 37°C constant *k* significantly increased from 50 ± 6.10^−4^ to 400 ± 46.10^−4^ after 3 h of incubation, respectively, and then gradually decreased to 135 ± 20.10^−4^ at 24 h of incubation period (Table 2). However, a substantial increase in the constant *k* was recorded at 70 and 100°C (from 30 ± 6.10^−4^ to 630 ± 81.10^−4^ and from 60 ± 15^−4^ to 860 ± 61^−4^, respectively) after 3 h of incubation, which later drastically decreased to 160 ± 18.10^−4^ and 160 ± 2.10^−4^ after 24 h incubation, respectively (Table 2). Our results indicated that the stabilities of azithromycin and tetracycline were temperature-dependent, and these antibiotics were relatively stable at 4 and 37°C during the 24 h time period (Tables 1 and 2). The obtained results were correlated with the good antibacterial activities of azithromycin and tetracycline at 4 and 37°C (Fig 3). However, no zone of inhibition was observed with *B*. *subtilis* when azithromycin in phosphate buffer was subjected to 70 and 100°C for 24 h which indicates that azithromycin lost its antibacterial activity when treated at 70 and 100°C for 24 h (Fig 3a), whereas the buffer containing tetracycline antibiotic subjected to the different temperature had a major effect on its antimicrobial activity. As the temperature of the buffer solution increased, decrease in the zone of inhibition was observed. Antibacterial activity of buffer solution after subjecting to different temperature with subsequent holding time period exhibited the differential inhibitory response against *B*.*subtilis* (Fig 3b). The results of present study were in agreement with results of previous reports, which showed that the heat treatment of cow milk or its processing into other milk products neither effectively eliminated nor reduced tetracycline residue [38–39]. Kitts et al. [40] studied the inactivation of oxytetracycline residues at a temperature range from 60 to100°C, and they recorded an increase in degradation rate in correlation with the increase in temperature and incubation time [40]. Similarly several researchers found that first order kinetics is an appropriate model for degradation study of β-lactams, quinolones, sulfonamides and tetracyclines [41–45]. β -lactam antibiotic such as penicillin and cephalosporins residues are commonly reported in milk as they are widely used veterinary drugs [46]. Thermal kinetics of β -lactam antibiotics was also observed to follow first order kinetic model and showed that, stability of β -lactam antibiotics is temperature dependent [43]. Further studies indicate that β -lactams can be significantly reduced in milk or water when subjected to 120°C for 15–20 min [43,47]. Macrolides such as erythromycin and azithromycin often used as an alternative to penicillin [48], are also known to be susceptible to heat treatment. Studies revealed that antimicrobial activity of erythtromycin was reduced when subjected to heat treatment [49]. However antimicrobial activity may not directly indicate the structural degradation of antibiotic but to date no sufficient kinetic data available for macrolides. Therefore in the present investigation we applied the degradation rate constant k to know the thermal kinetic of azithromycin, which was known to be an appropriate model for degradation study of antibiotics. Tetracycline is broad spectrum antibiotic most commonly used as veterinary drug and its residue is commonly reported in food produced from animal derivatives [50]. Various studies showed that tetracyclines are susceptible to heat treatment and degradation rate of tetracyclines depends upon the type of food matrix and cooking method. Oxytetracycline was highly susceptible to heat treatment when it was present in a food mitrix, buffer or water system [45,51]. Further the present study indicated that antibacterial activity of azithromycin and tetracycline were relatively stable at 4 and 37 °C even after 24 h, these results were in corroboration with the study of Lynda et al., reported that no loss of tetracycline was observed even after 48 h of storage at 4 °C and 24 h at 25 °C [52]. The results of present study on thermal kinetics of azithromycin and tetracycline would allow us to estimate the effect of sterilization and heating procedures on stability of antibiotic residues in milk and other food products.

### Effects of pH on the stability of azithromycin and tetracycline

The effects of different pH ranges on azithromycin stability were detected and shown in Table 3. A significant increase in azithromycin degradation rate constant *k* was detected at acidic pH of 4 and 5 (1820 ± 15.10^−4^ and 1010 ± 5.10^−4^, respectively) after 1 h of incubation, which later drastically decreased to 391 ± 2.10^−4^ and 221 ± 2.10^−4^, respectively, after 24 h incubation (Table 3). On the other hand, the constant *k* values of azithromycin were low at both pH of 8 and 9 at different incubation times (Table 3). The effects of pH on the stability of tetracycline were detected, and degradation rate constant *k* values were summarized in Table 4. At room temperature and at 1 h, the constant *k* of tetracycline was found to be 740 ± 82.10^−4^, 520 ± 53.10^−4^ and 50 ± 24.10^−4^ at pH of 9, 8 and 7 respectively, which then remarkably increased to 720 ± 34.10^−4^, 1420 ± 32.10^−4^ and 1170 ± 09.10^−4^ at pH of 6, 5 and 4, respectively. Similar changes were noted at all the time points where constant *k* values gradually increased with the decrease in pH level (Table 4). Likewise, Dehghani et al. (2013) demonstrate that the decrease in pH from 11 to 3 has a significant reduction in penicillin G stability, however the alkaline pH was more efficient for the antibiotic stability [53]. Similarly Loftin et al reported that stability and degradation pathway of tetracycline residues in food products largely depends on the pH of the food [54]. Kitts et al., found that the degradation rate of tetracycline compound was higher in buffer systems at different pH like 3 and 6.9 [40]. Whereas Xuan et al., showed that degradation of oxytetracycline in neutral solution was faster when compared with that of acidic or alkaline pH [55]. Similarly, tetracycline compounds showed increased degradation at higher temperature at neutral solution compared to acidic solutions [46]. Another antibiotics Lasalocid was stable in neutral and acidic pH but completely broken down when exposed to alkaline pH [56]. In addition, Seral et al. (2003) indicate that the acidic pH has an adverse effect on azithromycin stability with significant reduction in its antibacterial activity against *Staphylococcus aureus* [57]. Our results indicate that stability of antibiotics not only depend on temperature but also pH of the food has significant effect on the degradation of antibiotics.

**Table 3:** Azithromycine degradation constant rate *k* values at different pH levels.

“*k* (10^−4^)”						
Time (hour)	pH 4	pH 5	pH 6	pH 7	pH 8	pH 9
1	1820 ± 15^e^	1010 ± 5^e^	430 ± 4^d^	110 ± 2^e^	260 ± 2^e^	60 ± 0.6^e^
3	763 ± 7^d^	623 ± 2^cd^	333 ± 2^c^	80 ± 0.6^d^	160 ± 2^d^	13 ± 0.3^a^
6	678 ± 5^c^	578 ± 4^c^	366 ± 3^cd^	50 ± 0.4^c^	93 ± 0.8^c^	40 ± 0.2^d^
12	422 ± 2^ab^	344 ± 4^b^	215 ± 2^b^	15 ± 0.2^b^	58 ± 0.6^b^	30 ± 0.2^c^
24	391 ± 2^a^	221 ± 2^a^	134 ± 2^a^	7 ± 0.1^a^	31 ± 0.4^a^	20 ± 0.1^ab^

Different letters indicate statistically significant differences at *P* < 0.05 according to a Tukey’s honest significant difference *post hoc test*. Values are mean ± standard errors of triplicate samples.

**Table 4:** Tetracycline degradation constant rate *k* values at different pH levels.

“*k* (10^−4^)”						
Time in hours	pH 4	pH 5	pH 6	pH 7	pH 8	pH 9
1	1170 ± 09^e^	1420 ± 32^e^	720 ± 34^e^	50 ± 24^a^	520 ± 53^d^	740 ± 82^e^
3	856 ± 16^d^	633 ± 13^d^	373 ± 21^bc^	186 ± 23^de^	240 ± 21^c^	476 ± 62^d^
6	766 ± 12^c^	441 ± 26^c^	528 ± 42^d^	150 ± 34^cd^	170 ± 41^b^	315 ± 24^c^
12	497 ± 08^b^	310 ± 14^b^	298 ± 21^b^	115 ± 11^bc^	93 ± 32^a^	185 ± 21^ab^
24	354 ± 04^a^	265 ± 12^a^	197 ± 08^a^	94 ± 06^b^	86 ± 65^a^	116 ± 16^a^

Different letters indicate statistically significant differences at *P* < 0.05 according to a Tukey’s honest significant difference *post hoc test*. Values are mean ± standard errors of triplicate samples.

## Conclusion

The present study detected a high concentration of azithromycin and tetracycline residues in raw milk samples than prescribed limits by FDA, leading to inhibition of growth of *B*. *subtilis*. High temperatures of 70 and 100°C were sufficient to affect the stability and subsequent antibacterial activity of azithromycin. However, tetracycline was not completely eliminated at the same respective temperatures. Also, the acidic pH has shown a significant reduction effect on azithromycin and tetracycline in comparison with alkaline pH.

The detected antibiotics in the present study are at alarming level posing high risk to public health, inaddtion the study also revealed that the stability of antibiotics can be reduced by appropriate action in order to ensure safe drinking milk and milk products for consumers. Further the study concludes that screening of milk for antibiotics residues need to be strictly performed before it reaches the consumers as it will help to reduce the threat of residual contamination of the food chain.

### Compliance with Ethical Standards

All the authors ensure that manuscript complies with the Ethical Standards for this journal.

## Supporting information

S1 Fig Quantification of azithromycin and tetracycline standard antibiotics in HPLC based on their area counts and concentration. (TIF)
