# Supplementary figures and images for: Detection and determination of stability of the antibiotic residues in cow’s milk
Source: PLoS One. 2019 Oct 10;14(10):e0223475. doi: 10.1371/journal.pone.0223475 (PMC6786530; doi:10.1371/journal.pone.0223475)

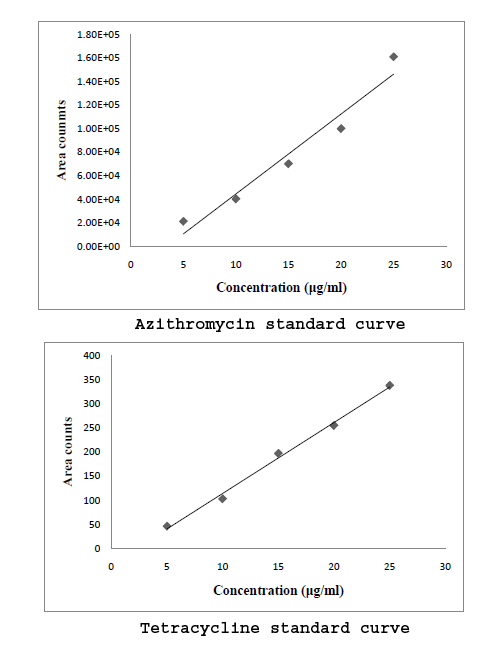

Supplement: S1 Fig — (TIF) [file pone.0223475.s001.tif]
